# Working against the clock: the impact of shift patterns on cognitive function, mental health, and stimulant use among healthcare workers

**DOI:** 10.1186/s12888-026-08246-z

**Published:** 2026-06-13

**Authors:** Jehad F. AlSamhori, Ansam Z. Baniamer, Hanen Batat, Abdelrahman M. Abu Sahyoun, Abdalrhman M. Hamasha, Mohammad Z. Alsharqwi, Abdel Rahman F. AlSamhori, Mohammad J. Al-Aqtash, Mohammad K. Al-Qurneh, Anas H. Khalifeh, Saif Alaribi

**Affiliations:** 1 School of Medicine, The University of Jordan, P.O.Box 11942, Amman, 11941 Jordan; 2 Faculty of Medicine, Yarmouk University, P.O.Box 566, Irbid, 21163 Jordan; 3 Jordan University Hospital, P.O.Box 13046, Amman, 11941 Jordan; 4 Department of Community and Mental Health Nursing, Faculty of Nursing, Zarqa University, P.O.Box 2000, Zarqa, 13110 Jordan; 5 Faculty of Medicine, Emirates International University, Sana’a, Yemen

**Keywords:** Shift patterns, Night shift, Mental health, Healthcare workers, Stimulant use

## Abstract

**Background:**

Shift work is common in healthcare and has been associated with negative physical, cognitive, and mental health consequences. It may also associate with greater dependency on stimulants as a way of overcoming fatigue and a lowered level of alertness.

**Aim:**

This study was to investigate an association between shift pattern, cognitive function, mental health (depression and anxiety), and stimulant use among healthcare workers.

**Methods:**

A descriptive cross-sectional design was used. A convenience sampling of 445 healthcare workers was recruited from two university-affiliated and two private hospitals in Jordan. Data was obtained from a self-administered questionnaire of sociodemographic characteristics, clinical symptoms, cognitive function, stimulant use, and validated scales.

**Results:**

A total of 445 healthcare workers were included (58.7% night shift, *n* = 261; 41.3% day shift, *n* = 184). Night shift workers reported significantly higher levels of fatigue (*n* = 200, 76.6%), headache (*n* = 164, 62.8%), and tremors (*n* = 114, 43.7%) and greater impairment in cognitive functions, especially memory (*n* = 173, 66.3%) and learning abilities (*n* = 156, 59.8%), than day shift workers (*p* < 0.05). Caffeine consumption and the use of stimulants were also considerably higher in the night shift workers. The mean depression score showed that the symptoms of depression were mild (*M* = 9.75, SD = 5.47) and the level of anxiety was moderate (*M* = 8.97, SD = 5.04). Stronger associations were found between depressive and anxiety symptoms and clinical symptoms and cognitive impairment among night shift workers.

**Conclusion:**

Night shift work was associated with more physical symptoms, cognitive impairment, and stimulant use and greater associations to depression and anxiety among healthcare workers. These results highlight the need for targeted interventions to ensure the safety of patients by attending to mental health, decreasing fatigue, and improving cognitive performance.

## Introduction

Healthcare systems around the world are increasingly dependent upon shift-based work schedules to provide around-the-clock care for patients, especially in hospital settings where care must be provided 24 hours a day. Around 15 million Americans are estimated to work nonstandard shifts [1]. Nonstandard work schedules, including rotating and night shifts, are characterized by sleep and interrupt sleep patterns and associated with adverse health outcomes. A person working shifts may even alternate from one type of shift to another every week or month [2]. While shift work has become an increasingly popular choice over the years, it is associated with greater physical and mental health risks and a decreased well-being that are usually related to circadian rhythm disturbance [3].

A growing body of evidence shows that shift work is associated with negative links to cognitive function, especially attention, memory, and executive functioning. Sleep deprivation and misalignment of the circadian rhythm are linked to damaged neurocognitive processes, which are associated with slowed reaction times, reduced alertness, and impaired decision-making abilities [4–8]. Studies among healthcare professionals have revealed that nights shift work is related to lower brain activity and lower cognitive functioning particularly in high-demand environments such as intensive care units [9, 10]. Moreover, systematic reviews have supported the presence of associates between shift work and impairments in working memory and attention, which are essential for safe clinical practice [8, 11].

Furthermore, healthcare workers exposed to an irregular schedule have associated it with negative mental health consequences. Researchers and healthcare organizations have noticed the increasing incidence of disorders such as depression, anxiety, and burnout affecting the healthcare professionals working on night shifts [3, 12, 13, 14]. Similar findings have been reported in Jordan where night shift work was associated to lower psychological well-being and disturbed sleep patterns among healthcare workers [15]. Furthermore, sleep disturbances due to shift work can also add to emotional dysregulation and cognitive decline and can associated with the further intensification of mental health issues [16–18].

Another emerging issue of concern is the growing dependence on the use of stimulants (caffeine, energy drinks, etc.) as a shift worker coping mechanism for fatigue and decreased alertness. Studies have shown that healthcare workers often consume stimulants to keep up their performance during the night shift work despite the possible negative association with their sleep and mental health [17, 19]. A literature review of the association of night shift work and nutrition patterns in nurses found a relatively consistent relationship between night work and increased coffee and caffeine intake along with other unhealthy behaviors, such as irregular eating, eating at night and decreased diet quality [20]. Excessive caffeine consumption has been associated with anxiety, sleep problems and depressive symptoms, which is a cycle of dependency associating with worsening health problems [21].

In the context of the Middle East and Jordan, studies on shift work have mainly focused on the physical health outcomes, such as cardiovascular diseases and metabolic syndrome [22]. However, more recent research has started exploring the sleep quality, mental health, and lifestyle behaviors in nurses and has found significant associations with shift work [15, 23, 24]. Although international or regional studies have examined the link of shift work to these outcomes separately, no research regionally or globally has simultaneously investigated the interconnected triad of cognitive impairment, declining mental health, and stimulant addiction among healthcare workers. Furthermore, previous Jordanian studies have examined these areas separately (sleep quality, mental health, and lifestyle behaviors), but none have assessed their combined correlation among healthcare workers. So, this study presents the integration, demonstrating how night shift work is linked to these overlapping risks. Understanding these combined is crucial for patient safety, as cognitive impairment, deteriorating mental health, and stimulant use together threaten clinical decision-making and performance. This comprehensive approach provides new evidence that previous, more focused studies failed to capture. Therefore, this study aims to examine the association between shift patterns, cognitive function, mental health, and stimulant use among healthcare workers.

## Methods

### Design, sampling, and settings

A descriptive, cross-sectional design was used. Healthcare workers were recruited through convenient sampling from four hospitals purposefully selected in Jordan to represent diverse healthcare settings (two university-affiliated hospitals and two private hospitals). These hospitals were chosen to ensure representation of geographical diversity (Amman and Irbid regions), hospital type (university-affiliated hospitals and private hospitals), and clinical settings serving diverse patient populations. The data was obtained during August and October of 2022. The inclusion criteria included working in clinical/intensive care departments and providing direct patient care as nurses, pharmacists, resident physicians, or specialist physicians with more than 6 months of experience. Also, participants were categorized into two groups: “night shift workers” if their primary work schedule included three or more night shifts per week and “day shift workers” if they worked only day shifts. This threshold was chosen to ensure continued exposure to night work, as repeated night shifts are likely to lead to clinically significant disruption of the circadian rhythm and sleep-wake inconsistency. Furthermore, this definition aligns with previous studies on shift work, which used several night shifts per week to denote regular or repeated night work [25].

Exclusion criteria included administrative/non-clinical staff, less than 6 months of experience, irregular shift work patterns, inability to complete questionnaires, and refusal to consent. Participants with irregular/rotating schedules (fewer than three regular night shifts per week) were excluded according to the inclusion criteria to ensure schedule homogeneity.

The sample size required was calculated with the use of Raosoft® [26]. Based on an estimated population of 17,258 healthcare providers in Jordan and assuming an expected frequency of 50% (a conservative estimate that increases the sample size), the minimum sample size required was 376 to achieve a 95% confidence level and a 5% margin of error (response distribution = 50%, accuracy = 5%).

### Study instruments

Data were collected through a self-administered English questionnaire designed to assess various aspects of sociodemographic characteristics, stimulant use, cognitive function, and mental health.

The sociodemographic characteristics, including age, gender, marital status, educational level, health profession, shift pattern, year of experience, salary ranges, and number of patients seen per shift. Additionally, the study considers chronic illnesses, medication use, headaches, fatigue, and tremors experienced during both night and day shifts. Also, Stimulant substance use, including cigarette, vapor, hookah usage alongside coffee, tea, energy drinks, alcohol, and multivitamins, with the mention of the general frequency of the consumption of each one.

In this study, cognitive function was assessed using short self-report questions that were incorporated into the questionnaire. These questions asked participants if they had problem with recalling information, learning new skills, and making decision during their regular clinical practice. In previous research with nurses and healthcare professionals, subjective cognitive was also employed [27, 28]. Rather than offering a complete neuropsychological profile, these measures were selected to highlight subjective cognitive concerns that are directly related to routine clinical performance. Standardized neurocognitive batteries were considered, but were not practical in the hospital settings where data were collected, as administering such batteries would have required substantial uninterrupted time per participant, risked disrupting clinical duties and patient care, and conflicted with variable shift schedules and staff availability. These items represent perceived changes in cognitive functioning and should be considered indicators of subjective cognitive difficulties rather than objective impairment.

Patient Health Questionnaire (PHQ-9), which is a nine-item instrument used for scoring the severity of depressive symptomatology where each item’s score is from 0 to 3 with a total range from 0 to 27, where minimal depression is 0–4, mild depression 5–9, moderate depression 10–14, moderately severe depression 15–19, and severe depression 20–27 [29]. The depression-adult scale’s severity measure demonstrated its internal consistency and reliability with a Cronbach’s alpha range between 0.68 and 0.94 [30].

Generalized Anxiety Disorder 7-item (GAD-7), which is a seven-item scale used for scoring the severity of anxiety symptomatology, where each item also scores from 0 to 3 with a total range from 0 to 21, where mild anxiety is 5–9, moderate anxiety 10–14, and severe anxiety 15–21 in total [31]. The internal consistency of the GAD-7 was good Cronbach’s alpha = 0.92 [31].

### Ethical considerations

This study was approved by the research ethical committee-Jordan University Hospital with the Institutional Review Board (IRB) approval number (ref.no. 5730). The Declaration of Helsinki 1964 ethical principles served as the guide for conducting this study. Participants who agreed to participate signed an informed consent form before receiving the study questionnaire. The subjects’ confidentiality and anonymity are protected. The survey was voluntary, and participants were free to withdraw from it at any time without incurring any consequences. All gathered information will be securely kept, and any soft copies will be password-protected

### Data collection procedures

Prior to data collection, ethical approval was obtained from the research ethical committee. The researcher coordinated with the managers of each selected hospital to explain the study’s purposes and the data collection method. Recruitment occurred during staff meetings, nursing handovers, and pharmacy shifts. The researchers distributed paper questionnaires to eligible participants during work breaks. In addition, the researcher assured that all participants are volunteering and willing to participate in the study and obtained consent from them.

### Data analysis

The data were analyzed using the Statistical Package for Social Sciences (SPSS), version 27 [32]. Descriptive analysis was obtained to describe sociodemographics, GAD-7, and PHQ-9. The normal distribution of PHQ-9 and GAD-7 was confirmed using Shapiro-Wilk tests (*p* > 0.05 for both scales). The analyses in this study were primarily exploratory and bivariate. Categorical variables were examined using chi-square and Fisher’s exact tests. All tests were two-tailed, and a *p*-value of 0.05 is considered significant.

## Result

### Participant characteristics

Of the 582 healthcare workers contacted, 445 completed the questionnaires (response rate 76.5%). As presented in Table 1, the average age of the study subjects was 29.97 years (SD = 6.08). Females were the majority of the sample (64.9%, *n* = 289). More than half (53.7%, *n* = 239) of the participants were married. Most of the participants had a bachelor’s degree (64.0%, *n* = 285). Concerning monthly income, most of the participants reported income between 500 to 1000 Jordanian Dinar (64.9%, *n* = 289).

**Table 1:** Participants demographic characteristics (*N* = 445)

Variables	N (%)
**Age, M=** 29.97; **SD=** 6.083	
**Gender**	
Male	156 (35.1%)
Female	289 (64.9%)
**Marital Status**	
Single	206 (46.3%)
Married	239 (53.7%)
**Education Level**	
High School Graduate, Diploma	43 (9.7%)
Bachelor’s Degree	285 (64.0%)
Master’s Degree	77 (17.3%)
Doctor’s Degree	40 (9.0%)
**Salary**	
<500 JDs	126 (28.3%)
500–1000 JDs	289 (64.9%)
>1000 JDs	30 (6.7%)
**Health Professionals**	
Residents	108 (24.3%)
Nurses	295 (66.3%)
Pharmacist	17 (3.8%)
Specialist	25 (5.6%)
**Shift Patterns**	
Day Shift Work (Shift A/B)	184 (41.3%)
Night Shift Work (Shift C)	261 (58.7%)
**Years of Work**	
<2 Years	159 (35.7%)
2–5 Years	90 (20.2%)
>5 Years	196 (44.0%)
**Patient Load per Shift**	
<10 Patients	85 (19.1%)
10–50 Patients	303 (68.1%)
50–100 Patients	45 (10.1%)
>100 Patients	12 (2.7%)
**Working Hours**	
<8 hours	11 (2.5%)
8 Hours	175 (39.3%)
12 Hours	186 (41.8%)
24 Hours	20 (4.5%)
Others	53 (11.9%)

*Note*. *M* = Mean; SD = Standard Deviation; *N* = Frequency; %=Percentage; JD = Jordanian Dinar

Table 1 indicated that nurses comprised the largest proportion (66.3%, *n* = 295) of the sample in terms of professional roles. The professional composition of the study sample (66.3% nurses) accurately reflects the reality of the Jordanian healthcare workforce, where nurses constitute between 65 and 70% of direct patient care providers. This high percentage of nurses underscores the importance of this study for the largest profession in Jordan’s healthcare sector.

More than half of the participants were night workers (58.7%, *n* = 261), and 41.3% (*n* = 184) were day workers. In the work experience, 44.0% (*n* = 196) had more than five years of experience. Most participants reported managing between 10 and 50 patients per shift (68.1%, *n* = 303). The most reported working shift length was 12 hours (41.8%, *n* = 186), and small proportions reported working less than 8 hours (2.5%, *n* = 11).

Clinical and stimulant history is shown in Table 3. Most participants did not have any chronic diseases (*n* = 406, 90.2%). Also, most of the participants reported no regular medication (*n* = 379, 85.2%), while 11.7% (*n* = 52) reported the intake of painkillers. Regarding work-related symptoms, more than half of the study population reported headaches during shifts (*n* = 260, 58.4%), and fatigue was reported by a large majority (*n* = 314, 70.6%). 34.6% (*n* = 154) of the participants reported experiencing tremors. In terms of cognitive function, 58.4% (*n* = 260) reported a negative link of shift work on their ability to remember information, 55.3% (*n* = 246) reported a negative link on learning new skills, and 47.6% (*n* = 212) reported impaired decision-making ability.

**Table 2:** Level of anxiety and depression

Variable	N	%
**Depression Total Score; M=**9.75, **SD** = 5.47		
**Category**		
Minimal	78	17.5
Mild	152	34.2
Moderate	125	28.1
Moderately Severe	71	16.0
Severe	19	4.3
**Anxiety Total Score; M=**8.97, **SD** = 5.04		
**Category**		
Mild	133	29.9
Moderate	144	32.4
Moderately Severe	112	25.2
Severe	56	12.6

*Note*. *M* = Mean; SD = Standard Deviation; *N* = Frequency; %=Percentage

Caffeine intake was common among participants, with 53.5% (=238) reporting intake of less than three cups per day and 23.8% (=106) consuming 3–5 cups daily. Regarding the stimulant substance intake frequency, 42.0% (*n* = 187) used stimulants once per day. Also, the majority of the participants were non-smokers (*n* = 363, 81.6%), and other smaller proportions reported different levels of cigarette consumption. Similarly, the majority did not use e-cigarettes (*n* = 381, 85.6%) or hookah (*n* = 379, 85.2%), as presented in Table 3.

### Anxiety and depression level

Table 2 shows that the mean depression score for the participants was 9.75 (SD = 5.47), indicating overall mild depressive symptoms. Specifically, 34.2% of participants (*n* = 152) were mildly depressed, 28.1% (*n* = 125) were moderately depressed, 16.0% (*n* = 71) were moderately severely depressed, and 4.3% (*n* = 19) were severely depressed. The mean anxiety score was 8.97 (SD = 5.04), indicating a moderate level of anxiety. About 29.9% (*n* = 133) had mild anxiety, 32.4% (*n* = 144) moderate anxiety, 25.2% (*n* = 112) moderately severe anxiety, and 12.6% (*n* = 56) severe anxiety.

**Table 3:** The differences of clinical history, cognitive function, stimulant substance history based on shift pattens

Variable	N (%)	Shift Patterns		P
		Day Shift N (%)	Night Shift N (%)	
**Clinical History**				
**Chronic Diseases**				
No Chronic Diseases	406 (90.2%)	164 (89.1%)	242 (92.7%)	0.004*
Diabetes Mellitus	7 (1.6%)	4 (2.2%)	3 (1.1%)	
Hypertension	12 (2.7%)	11 (6.0%)	1 (0.4%)	
Respiratory Disorders	11 (2.4%)	3 (1.6%)	8 (3.1%)	
Back Pain	5 (1.1%)	1 (0.5%)	4 (1.5%)	
Migraine	3 (0.7%)	1 (0.5%)	2 (0.8%)	
Stroke	4 (0.9%)	1 (0.5%)	3 (1.1%)	
Gout	1 (0.2%)	1 (0.5%)	0 (0.0%)	
Iron deficiency anemia	1 (0.2%)	1 (0.5%)	0 (0.0%)	
**Medication Usage**				
No Medicine Intake	379 (85.2%)	161 (87.5%)	218 (83.5%)	0.648
Selective serotonin reuptake inhibitor	1 (0.2%)	0 (0.0%)	1 (0.4%)	
Aspirin	4 (0.9%)	2 (1.1%)	2 (0.8%)	
Pain Killers	52 (11.7%)	17 (9.2%)	35 (13.4%)	
Beta-Blockers	2 (0.4%)	1 (0.5%)	1 (0.4%)	
Vitamin Supplements	2 (0.4%)	1 (0.5%)	1 (0.4%)	
Proton-pump inhibitor	1 (0.2%)	1 (0.5%)	0 (0.0%)	
Thyroxine	4 (0.9%)	1 (0.5%)	3 (1.1%)	
**Headache through the Shift**^**a**^				
Yes	260 (58.4%)	96 (52.2%)	164 (62.8%)	0.005*
No	72 (16.2%)	42 (22.8%)	30 (11.5%)	
Sometimes	113 (25.4%)	46 (25.0%)	67 (25.7%)	
**Fatigue through the Shift**^**a**^				
Yes	314 (70.6%)	114 (62.0%)	200 (76.6%)	<0.001**
No	57 (12.8%)	35 (19.0%)	22 (8.5%)	
Sometimes	74 (16.6%)	35 (19.0%)	39 (14.9%)	
**Tremors through the Shift**^**a**^				
Yes	154 (34.6%)	40 (21.7%)	114 (43.7%)	<0.001**
No	201 (45.2%)	99 (53.8%)	102 (39.1%)	
Sometimes	90 (20.2%)	45 (24.5%)	45 (17.2%)	
**Cognitive Function**				
**Impact on Recalling Information**^**a**^				
Yes	260 (58.4%)	87 (47.3%)	173 (66.3%)	<0.001**
No	94 (21.1%)	50 (27.2%)	44 (16.9%)	
Sometimes	91 (20.4%)	47 (25.5%)	44 (16.9%)	
**Impact on the Ability to Learn New Skills**^**a**^				
Yes	246 (55.3%)	90 (48.9%)	156 (59.8%)	<0.001**
No	151 (33.9%)	83 (45.1%)	68 (26.1%)	
Sometimes	48 (10.8%)	11 (6.0%)	37 (14.1%)	
**Impact on Decision Making**^**a**^				
Yes	212 (47.6%)	85 (46.2%)	127 (48.7%)	0.276
No	135 (30.3%)	63 (34.2%)	72 (27.6%)	
Sometimes	98 (22.0%)	36 (19.6%)	62 (23.8%)	
**Stimulant Substance History**				
**Caffeine Intake**^**a**^				
No	71 (16.0%)	46 (25.0%)	25 (9.6%)	<0.001**
Less than 3 cups	238 (53.5%)	103 (56.0%)	135 (51.7%)	
3–5	106 (23.8%)	24 (13.0%)	82 (31.4%)	
More than 5 cups	30 (6.7%)	11 (6.0%)	19 (7.3%)	
**Type of Stimulation used**^**a**^				
Tea	192 (37.9%)	82 (44.6%)	110 (42.1%)	0.706
Coffee	15 (3.0%)	6 (3.3%)	9 (3.4%)	
Energy Drink	93 (18.3%)	35 (19.0%)	58 (22.2%)	
Alcohol	4 (0.8%)	2 (1.1%)	2 (0.8%)	
Multi-Vitamin	30 (5.9%)	14 (7.6%)	16 (6.1%)	
No	173 (34.1%)	65 (35.3%)	108 (41.4%)	
**Stimulation Substance Intake Per Day**^**a**^				
No	157 (35.3%)	59 (32.1%)	98 (37.5%)	0.017*
1	187 (42.0%)	92 (50.0%)	95 (36.4%)	
2	64 (14.4%)	18 (9.8%)	46 (17.6%)	
3	25 (5.6%)	12 (6.5%)	13 (5.0%)	
4	12 (2.7%)	3 (1.6%)	9 (3.4%)	
**Cigarette usage**^**a**^				
No	363 (81.6%)	154 (83.7%)	209 (80.1%)	0.192
Few Cigarettes	18 (4.0%)	9 (4.9%)	9 (3.4%)	
Less than one Package	23 (5.2%)	11 (6.0%)	12 (4.6%)	
1–2 Packages	31 (7.0%)	8 (4.3%)	23 (8.8%)	
More than 2 Packages	10 (2.2%)	2 (1.1%)	8 (3.1%)	
**E-Cigarettes or Vapor usage**^**b**^				
No	381 (85.6%)	157 (85.3%)	224 (85.8%)	0.992
1 Filling	44 (9.9%)	19 (10.3%)	25 (9.6%)	
2 Fillings	10 (2.2%)	4 (2.2%)	6 (2.3%)	
3 and more Fillings	10 (2.2%)	4 (2.2%)	6 (2.3%)	
**Hookah**^**b**^				
No	379 (85.2%)	150 (82.0%)	228 (87.4%)	0.033*
1–2 a week	31 (7.0%)	11 (6.0%)	20 (7.7%)	
3 and more a week	8 (1.8%)	6 (3.3%)	2 (0.8%)	
Almost every day	17 (3.8%)	12 (6.6%)	5 (1.9%)	
One a month	10 (2.2%)	4 (2.2%)	6 (2.3%)	

*Note*. *N* = Frequency; %=Percentage; *χ*^2^ = chi-square; a=chi-square; b = Freeman–Halton exact test; *=*P*<0.05; **=*P*<0.001

### The differences of clinical history, cognitive function, and stimulant history based on shift patterns

The clinical history, cognitive function, and stimulant history differences between the shift patterns were examined using Chi-square and Fisher Exact test. Significant differences were found between the day and night shift workers in a number of variables, as presented in Table 3. The chi-square test shows significant night shift workers complained of higher rates of headache, fatigue, and tremors. In terms of cognitive function, night shift workers were significantly more likely to report negative associations with their ability to recall information and learn new skills. There was, however, no significant difference found between groups in decision-making ability. Regarding stimulant use, the chi-square test shows significant differences in caffeine intake and frequency of stimulant use per day, with night shift workers having higher levels of consumption. Also, a Fisher’s exact test indicates that Hookah use significantly differed between shift patterns, while cigarette and e-cigarette use did not have statistically significant differences.

### The association depressive and anxiety symptoms with clinical history and cognitive function among shift patterns

Associations between depressive and anxiety symptoms and clinical history and cognitive function were investigated in the day and night shift healthcare workers using Chi-square and Fisher’s exact tests, as present in Tables 4 and 5. Among the day shift workers, depressive symptoms were significantly correlated with poor cognitive functioning, especially recall of information, ability to learn new skills, and ability to make decisions. The greatest levels of impairment were among the population experiencing moderate to moderately severe depression. Fatigue and tremors were also found to have significant relationships with depressive symptoms, while headache did not show a significant relationship. Among the night shift workers, depressive symptoms showed stronger associations. Significant relationships were found with learning new skills, ability to make decisions, headache, fatigue, and tremors. The highest impairment and symptom rate was consistently found in those who had severe or moderately severe depression. However, there was no significant association with information recall, as presented in Table 4.

**Table 4:** The association level of depressive symptoms with clinical history and cognitive function among shift patterns

Variable	Shift Patterns											
	Day Shift						Night Shift					
	Minimal	Mild	Moderate	Moderately Severe	Severe	P	Minimal	Mild	Moderate	Moderately Severe	Severe	P
**Impact on Recalling Information**	17.1%	50.8%	**68.8%**	47.8%	42.9%	^a^<0.001**	59.5%	58.6%	76.6%	68.8%	66.7%	^a^0.053
**Impact on the Ability to Learn New Skills**	34.1%	40.0%	60.4%	**78.3%**	42.9%	^b^<0.001**	40.5%	54.0%	72.7%	56.3%	**91.7%**	^a^<0.001**
**Impact on Decision Making**	31.7%	43.1%	**60.4%**	47.8%	57.1%	^a^<0.001**	35.1%	40.2%	62.3%	45.8%	**75.0%**	^a^<0.001**
**Headache through the Shift**	41.5%	60.0%	54.2%	47.8%	42.9%	^a^0.437	35.1%	63.2%	63.6%	75.0%	**91.7%**	^a^<0.001**
**Fatigue through the Shift**	41.5%	63.1%	72.9%	**82.6%**	28.6%	^a^<0.001**	43.2%	79.3%	83.1%	**85.4%**	83.3%	^a^<0.001**
**Tremors through the Shift**	4.9%	21.5%	33.3%	21.7%	**42.9%**	^a^0.003*	24.3%	39.1%	48.1%	50.0%	**83.3%**	^a^<0.001**

*Note*. *N* = Frequency; %=Percentage; *χ*^2^ = chi-square; a=chi-square; b = Freeman–Halton exact test; *=*P*<0.05; **=*P*<0.001

**Table 5:** The association level of anxiety symptoms with clinical history and cognitive function among shift patterns

Variable	Shift Patterns										
	Day Shift						Night Shift				
	Mild	Moderate	Moderately Severe	Severe	P	Mild		Moderate	Moderately Severe	Severe	P
**Impact on Recalling Information**	29.8%	48.5%	**65.9%**	55.6%	^a^0.003*	53.9%		67.1%	71.8%	**78.9%**	^a^0.033*
**Impact on the Ability to Learn New Skills**	45.6%	45.6%	56.1%	55.6%	^b^0.586	51.3%		48.7%	71.8%	**76.3%**	^a^<0.001**
**Impact on Decision Making**	31.6%	41.2%	**68.3%**	61.6%	^a^0.003*	44.7%		39.5%	57.7%	**57.9%**	^a^0.017*
**Headache through the Shift**	56.1%	47.1%	48.8%	66.7%	^a^0.681	50.0%		53.9%	73.2%	**86.8%**	^a^<0.001**
**Fatigue through the Shift**	45.6%	69.1%	63.4%	**83.3%**	^a^0.027*	71.1%		72.4%	78.9%	**92.1%**	^a^0.024*
**Tremors through the Shift**	8.8%	22.1%	36.6%	27.8%	^a^0.064	19.7%		53.9%	43.7%	**71.1%**	^a^<0.001**

*Note*. *N* = Frequency; %=Percentage; *χ*^2^ = chi-square; a=chi-square; b = Freeman–Halton exact test; *=*P*<0.05; **=*P*<0.001

Regarding the symptoms of anxiety, Table 5 showed a significant association among day shift workers to recall of information, decision-making ability, and fatigue, with higher levels of impairment being associated with moderate to severe anxiety. No significant relationships were found with learning new skills, headaches, and tremors. In contrast, in the night shift workers, anxiety symptoms were associated more strongly among variables. Significant relationships were found with recall of information, learning new skills, ability to make decisions, headache, fatigue, and tremors, with the highest levels of impairment found in participants with severe anxiety.

## Discussion

This study investigated the relationship between shift work and cognitive function, mental health, and stimulant use among healthcare workers. The current study has shown that the healthcare workers experienced mild levels of depression and moderate levels of anxiety, and a significant proportion of the participants reported moderate to severe symptoms. These results are in line with the literature showing that healthcare workers, especially those working under stressful work requirements and shift work, are more likely to be at risk of psychological distress. For example, research has shown that shift workers have elevated levels of depression and anxiety over non-shift workers because of circadian rhythm disruption, sleep deprivation, and occupational stress [3, 14]. Similarly, recent evidence among healthcare professionals shows that night shift duties are significantly associated with insomnia, fatigue and poorer mental health outcomes [13, 33, 34]. In the Jordanian context, Batat et al. [15] concluded that night shift work was significantly associated to poor psychological well-being and mental health among healthcare workers. In addition, Anan et al. [35] reported high levels of psychological distress in Jordanian nurses, especially in critical care areas, and suggested that occupational demands correlated with emotional burden.

However, the relative mild level of depression found in this study seems to be lower compared to other international studies with higher findings of prevalence of severe depressive symptoms among healthcare workers (Afif Wahyudi, 2025 [35, 36]). This discrepancy may be explained by contextual factors such as cultural coping mechanisms, social support systems, or work environment differences. It is also possible that the relatively young sample may show greater psychological resilience than older populations [16]. Also, the prevalence of mild depression presents a challenge to severe cases elsewhere (Afif Wahyudi, 2025), possibly due to a lack of self-reporting, cultural adaptation, or support in Jordan something that cannot be generalized without longitudinal data.

The findings of this study showed significant differences between the day and night shift workers in terms of clinical symptoms and cognitive function and stimulant use, with the night shift workers reporting higher levels of headache, fatigue, tremors, cognitive impairment, and stimulant consumption. In our study, we found that night shift workers are less likely to have chronic illness than day shift workers. The youthful mean age of our respondents probably had a significant association with our findings. Additionally, diabetes is more likely to be present in the day shift team than in the night shift team. Kroenke et al. [37] disagreed with the links between translocation and diabetes through glucose dysregulation. These differences may be attributable to the increased serum glucose [38] and body mass [39] associated with night shifts. Furthermore, night shift workers are more likely to develop moderate to severe asthma than day shift workers, which may be due to a mismatch between the internal body clock and the environment [40]. These results are supported by present findings showing that night shift work is characterized by circadian rhythms and sleep patterns that associated to increased fatigue and physical symptoms [15, 33, 41–43]. Previous research has also reported that the prevalence of headaches, fatigue, and somatic complaints is higher in night shift workers than in day workers [4, 7, 8, 10, 33, 43–45].

Regarding cognitive function, the study finding that night shift workers had higher levels of impairment in learning new skills and remembering information is consistent with other research that shows sleep deprivation and circadian misalignment have a negative association with memory, attention, and executive functioning [4, 7, 8, 46]. This conclusion is further supported by systematic reviews that have shown that shift work is related to lower executive functions, memory/learning, attention, and perceptual-motor function [5, 11]. Despite the overall consistency, some findings depart from previous studies. Notably, this study did not find any significant difference between the day and night shift workers in terms of decision-making ability, although previous research frequently reported impairments in executive functions, including decision-making, in night shift workers [6]. This may be due to the protocol-driven and collaborative nature of Jordanian hospital environments, which reduces the individual cognitive burden, unlike more independent work environments [5]. Our younger cohort (mean age 30 years) is more likely to demonstrate resilience, whereas older individuals with changing jobs tend to decline more rapidly; moreover, self-reporting may underestimate minor deficiencies without objective testing.

The higher levels of stimulant use, especially caffeine consumption by night shift workers, are also consistent with previous findings. Buchvold et al. [47] found a significant positive association between night shifts and daily caffeine consumption, which could indicate that caffeine was being used as a stimulant during work at night. Healthcare workers regularly use stimulants to keep them awake and alert for those long night shifts [17]. Misalignment with the biological clock leads to increased fatigue through adenosine buildup, prompting the use of caffeine/stimulants to block receptors and enhance alertness [17, 47]. Hookah use has increased among night shift workers, but not cigarettes, which differs from the study by Buchvold et al. [47]; cultural norms in the Middle East prioritize hookah, indicating social and cultural overrides of global shift effects. In Jordan context, the highest smoking prevalence was reported in nurses who work predominately night shift [24]. This may be accounted for by cultural, especially Middle Eastern, wherein alternative forms of tobacco use such as hookah prevalent than cigarette use. This interpretation is supported by the results, suggesting that the type of stimulant or substance use may vary according to sociocultural norms as opposed to shift work solely.

In terms of mental health, the present study showed significant links between depressive symptoms and poor cognitive and clinical outcomes in shift workers. These findings are consistent with the previous literature, which shows that depression is associated with strong deficits in memory, learning, and executive functioning [48, 49]. Network-based analyses among healthcare workers indicate that depressive symptoms, particularly those related to low motivation, concentration difficulties, and mental slowing, are closely associated with cognitive deficits such as difficulties in recalling information, difficulty in initiating tasks, and attention [50]. Similarly, Yeo et al. [51] found that depression in shift workers is strongly associated to fatigue, poor sleep quality, and psychological stress, all of which have an indirect relation to decreased cognitive performance. Moreover, associations that are found among night shift workers are consistent with studies that suggest that shift work is a source of psychological distress because of sleep disruption, occupational stress, and circadian misalignment [3, 41].

The results also showed depressive symptoms were related to fatigue and tremors, which is consistent with previous evidence that depression is related to somatic symptoms and physical exhaustion [52]. However, there is no significant association of depression and headaches in day shift workers. A possible explanation for this is that headaches among day workers may be determined more by occupational or environmental factors than by psychological distress alone. An important inconsistency was the lack of a significant relationship between the depressive symptoms and information recall among the night shift workers, despite the strong associations reported in previous research [49]. However, chronic consumption disrupts the balance of the hypothalamic-pituitary-adrenal axis, leading to persistent anxiety and depression: our data show stronger associations among night shift workers (e.g., severe anxiety is associated with all cognitive and somatic symptoms). Sleep deprivation impairs prefrontal cortex networks (memory/learning), and this is exacerbated by stimuli tolerance, which causes cycles of tremor/fatigue explaining the lack of association between memory and depression [11].

Regarding the issue of anxiety, study results showed higher and broader associations to cognitive and clinical impairment in the night shift workers compared with day workers. These results are consistent with previous studies showing that anxiety has a negative association on the working memory, attention, and decision-making processes [52, 53]. Additionally, the general association of anxiety in night shift workers is consistent with the idea that circadian disruption increases vulnerability of psychology and its link to function [17]. In contrast, the limited associations between anxiety and some variables (e.g., learning new skills, headache, and tremors) among day shift workers are different from studies of possible more generalized cognitive impairment in anxious individuals. This discrepancy may be explained by the lower levels of physiological stress and better sleep quality in the day shift workers which could buffer the link of anxiety on cognitive performance.

### Implications

Night work among healthcare workers contributes to increased fatigue, impaired cognition, and stimulant dependence, risking their occupational safety through increased errors and the emergence of physical symptoms such as tremors. These effects emphasize the necessity of strategies to mitigate the risks associated with night work in high-risk clinical settings. Furthermore, the cognitive decline in memory and decision-making resulting from night work directly threatens the quality of patient care, as reduced alertness increases the likelihood of medication and procedure errors. The strong correlation between mental health problems and clinical symptoms among night workers exacerbates these risks, underscoring the need to address the impact of night work on performance. These results have significant implications for clinical practice, regular mental health screenings are essential, particularly for night shift workers, to enable early detection of depression and anxiety before they negatively impact cognitive function. Integrating these screenings into routine occupational health checks, along with education on how to recognize symptoms, supports proactive intervention and enhances overall employee resilience. Early identification of depression and anxiety can help prevent further cognitive decline and make patients safer. Additionally, workplace wellness programs should include encouraging healthy coping mechanisms and limiting the use of stimulants like caffeine.

From an administrative perspective, healthcare institutions should consider ways to optimize shift schedules, limit prolonged night shifts, and implement fatigue management strategies. Providing structured breaks, mental health support services, and education on sleep hygiene may help to reduce the negative association of shift work. In addition, the incorporation of supportive work environments and teamwork-based approaches can help reduce the cognitive burden and improve decision-making performance. Future research should utilize longitudinal and interventional study designs to better understand causality and evaluate strategies for mitigating the effects of shift work. Exploring moderate factors such as resilience, coping mechanisms, and organizational support are also recommended.

### Strengths and limitations

This study has several strengths. It included a relatively large and diverse sample of healthcare workers from two university-affiliated hospitals and two private hospitals. The study also used validated and well-known instruments. Additionally, the study assessed different dimensions at the same time, such as cognitive function, clinical symptoms, and stimulant use, which gave a complete understanding of the relation of shift work.

However, there are several limitations to consider. The cross-sectional design identifies correlations but excludes proving causality or temporal relationships. The present study was conducted in a limited number of hospitals in Jordan which might affect the generalizability of the results. Also, a convenience sample was used which may cause selection bias and limit the generalizability of the findings. The sample included a very high number of nurses, reflecting the structure of the Jordanian healthcare workforce, although the results may not completely represent other professional groups or healthcare settings.

Data were gathered with self-reported questionnaires, which may be prone to recall bias and social desirability bias. Another limitation of this study was the use of self-report questions to measure cognitive function rather than standardized neuropsychological testing. Subjective cognitive complaints are sensitive to recall and response bias. Objective testing of cognition would have provided a more complete assessment but was not possible in this hospital-based study due to time restrictions, shift overlap, and the need to avoid interfering with patient care.

In addition, statistical limitations include reliance exploratory and based on bivariate tests, without multivariable adjustment for potential confounders or corrections for multiple comparisons, which may increase the risk of type I error. As a result, the relationships identified between shift pattern and outcomes may be influenced by measured or unmeasured characteristics such as age, occupation, sleep duration, workload, department type, years of experience and chronic disease. Because of this, it is impossible to completely isolate the independent impact of working nights. Another limitation of the study is that a number of occupational factors, such as validated sleep quality, burnout, work intensity, staffing ratios, and the frequency of consecutive night shifts, that may have an impact on mental health and cognitive performance were not evaluated. The association between shift work and the observed effects may be confounded or mediated by several factors.

Therefore, to enhance the accuracy, comparability, representativeness, and external validity of the assessment, future research should use standardized neurocognitive tools in conjunction with self-report measures, validated sleep and burnout scales, detailed shift scheduling information, and objective workload indicators, using probabilistic sampling techniques, multivariate regression models, or other advanced analytical methods to improve the characterization of occupational exposure, adjust for confounding variables, and elucidate the independent impact of shift work on cognitive functions, mental health, and stimulant use outcomes.

## Conclusion

This study highlights that among healthcare workers, night shift work is linked to increased levels of fatigue, cognitive impairment, and increased stimulant use, and moderate levels of depression and anxiety. Night shift workers showed more susceptibility to adverse clinical and cognitive associations than day shift workers. These findings highlight the importance of specific interventions to promote the mental health and cognitive functioning of healthcare workers, especially those working on the night shift, to maintain the well-being of healthcare workers and patient safety.

## Data Availability

The data that support the findings of this study are available on request from the corresponding author.
